# A holistic view of maritime navigation accidents and risk indicators: examining IMO reports from 2011 to 2021

**DOI:** 10.1186/s41072-023-00135-y

**Published:** 2023-04-23

**Authors:** Carine Dominguez-Péry, Rana Tassabehji, Franck Corset, Zainab Chreim

**Affiliations:** 1 University Grenoble Alpes, Grenoble INP (Institute of Engineering and Management), CERAG, 38000 Grenoble, France; 2 School of Management, Bath University, Bath, BA2 7AY UK; 3 University Grenoble Alpes, LJK, 38000 Grenoble, France

**Keywords:** IMO accident reports, Maritime ecosystem, IRAMUTEQ, Risk indicators

## Abstract

This paper investigated the risk indicators in maritime accidents and how they are considered within the reporting of maritime accidents, drawing on ten years of International Maritime Organisation (IMO) (2011–2020) accident reports. It highlighted the lack of consistent findings in studies exploring the role of vessel characteristics in maritime accidents, which often result from different methods, databases, techniques and motivations used by each respective study for gathering and analysing data. Furthermore, as human error continues to be highlighted as the top-cited cause of accidents, this study examined the qualitative content of IMO accident reports in-depth to broaden our understanding of maritime accident risk factors. Using a data-driven approach, statistical (ANOVA) and advanced text-mining techniques (using IRAMUTEQ software) were applied to extract meaning from the semi-structured and unstructured narrative descriptions that constitute most of the national administrations’ investigation reports to the IMO. Building on the text analysis of the IMO accident data, we proposed the Accident Maritime Ecosystem framework, which incorporates individuals, the ship organisation (on board), the internal ship ecosystem (on board and onshore), the external ship ecosystem (external factors) and the global maritime ecosystem (policies and regulations); moreover, it identifies these entities as risk factors in maritime accidents. The findings illustrate how accident reporting is largely human-centric and that as maritime transportation is becoming increasingly complex, there is a need for policy and organisational decision-makers to incorporate a broader scope of actors when considering maritime risk factors, which can be achieved by using the AME framework as a guideline.

## Introduction

The COVID-19 pandemic directly impacted the maritime transportation sector with serious ramifications for global supply chains. This was perfectly illustrated in the case of the accident involving the MV Ever Given, which played out in the full glare of the public around the world. The 220,000 dwt ton container ship, carrying billions of dollars’ worth of cargo, ran aground in the Suez canal in March 2021 during high winds and poor weather (Guardian 2021a). This massive container ship blocked the world’s busiest marine waterway for six days, creating a massive maritime traffic jam and further disrupting the already stressed supply chains and shipping logistics hit by the Coronavirus pandemic. This single incident is estimated to have cost global trade between $6 and $10 billion daily (Guardian 2021b). Despite the strong winds and sandstorms causing poor visibility and difficulty in navigation, reports highlighted human error (HE) (navigational/steering mistakes by the Captain and/or the pilots) as playing a key role in the accident (Khanna 2021; WSJ 2021; WP 2021). This is consistent with many studies into maritime accidents, where HE is often identified as one of the primary factors in over 75% of maritime accidents (Acejo et al. 2018; Celik and Cebi 2009). However, questions have been raised about the origins and reliability of the ‘80% human error impact on maritime safety’ ‘myth’ (Wrobel 2021). Furthermore, because of the political, commercial, financial and legal implications of apportioning blame for accidents, it remains unclear exactly what constitutes HE in official reports (Yim 2017). Here, we posit that attributing ‘human factors’ as the leading cause of accidents prevents a full understanding and appreciation of the complex nature of associated risk indicators that might remain unknown contributing factors. Ultimately, this will make it even more difficult to address and improve maritime safety.

The secretary-general of the International Chamber of Shipping highlighted the problem of focusing mainly on the HE role in maritime accidents. He was increasingly concerned that the ‘complex series of events’ that cause accidents at sea often result in blaming HE and the criminalisation of seafarers (George 2021). However, taking the example of the Ever Given, one maritime historian argued that “*if it’s the wind or the weather, that’s human because …we have the ability to detect the wind. If they knew high winds were a potential that morning why bring the ship into the canal?*” (George 2021). Thus, there is a need to understand better the complexity of reported maritime accidents and their different and interrelated causes.

This paper argues that how the industry and scholars consider HE as being central to accidents may obscure major issues in the broader ships’ environment, such as the ship’s organisational and inter-organisational management extending across the maritime ecosystem (that includes ship owners, ports, insurers, classification, and international maritime organisations). This might also hinder improvements in safety, training, and management of the ship organisation and relationships between the ship organisation and her direct ecosystem.

Thus, the principal aim of this study is to go beyond a human-centric focus on the causes of maritime accidents and take a broader, more holistic look at the risk indicators that might contribute to maritime accidents. The objectives are (i) to identify and classify the risk indicators in maritime accident investigation reports submitted to the IMO (International Maritime Organisation) for ten years, (ii) to demonstrate statistical and advanced text mining techniques to extract meaning from the qualitative narratives of the IMO accident reports and (iii) to define and conceptualise the broader scope of risk indicators for maritime accidents.

This paper will first provide a brief overview of the literature on the causes of maritime accidents and how they are reported. It will then present the methodology, namely a quantitative statistical approach to analyse maritime accidents at the macro-level related to the severity of accidents by the number of deaths and characteristics of maritime vessels involved in the accidents. The second part of the analysis will use text mining techniques (IRAMUTEQ) to improve our understanding of maritime accidents by analysing the content of accident reports by drawing out and categorising the causes of maritime accidents beyond the predominant individual and human error. Finally, the paper will include a discussion, make recommendations and highlight the limitations of this study.

## Enlarging the scope of causes of maritime accidents: moving beyond the narrow focus on human error

HE is often cited as the main contributing factor in maritime accidents. However, Wrobel (2021) was unable to find the ‘origins of the mythical 80% involvement of human error in maritime accidents’ in his review of the published literature. Indeed, most studies use HE as a catch-all term but do not offer further precision (Zhang et al. 2019b). Scholars agree that the terms ‘human error’ and ‘human factors’ are generally ill-defined, with little distinction between them and are often used interchangeably (Dominguez-Péry et al. 2021a). Arguably, identifying the causes of accidents is largely dependent on the discipline and the perspective of the entities evaluating them (Dominguez-Péry et al. 2021a). For instance, from the engineering perspective, HE is considered a variable that must be tackled to avoid accidents. For ergonomics, HE is more complex; it includes factors of an organisational nature but has no systematic solutions to solve the causes. From a management/operations perspective, HEs are embedded in human tasks as they interact with systems or technologies and the related risks to improve human reliability. Furthermore, many studies have attempted to classify accidents because HE based on unsafe operations (Shappel and Wiegmann 1997), operator error, and system deficiencies (Patterson and Shappell 2010). This is problematic because, over the decades, the human role in the maritime context has evolved; thus, HE incorporates an increasingly complex interaction between people, tools, advanced technologies, and tasks in an organisational environment that extends beyond the boundaries on board and onshore (Dominguez-Péry et al. 2021a and b).

By analysing the investigation manuals of eight accidents in different industries, Lundberg et al. (2009) found that all the manuals focus on human, technological, organisational, and information factors with linear complex models; as a result, analyses tend not to consider accidents holistically. In order to avoid a fragmented understanding of accidents and improve safety, Hollnagel (2020) proposes a unification or ‘synesis’ approach incorporating productivity, quality, reliability and safety (Fig. 6.1, p.81) and integrating the understanding of factors (design, organisation, maintenance, technology) before (‘upstream’) and after (‘downstream’) the accident. However, this approach does not seem to have significantly impacted accident and safety theory or practice, as most of the literature still focuses on individual factors as causes of maritime accidents. For instance, individual fatigue (Stratmann and Boll 2016), situation awareness (Grech et al. 2002; Razavi et al. 2014; Stratmann and Boll 2016), slip or skill/knowledge deficiencies (Yim 2017) and very rarely labour conditions (Wang et al. 2021), unsafe supervision (Shi et al. 2021), seafarer competence/skills (Yim 2017; Wang and Yin 2020), and at the team level individual crew error (Changhai and Shenping 2019). Only a very few studies are beginning to consider errors at the organisational level (Bhardwaj et al. 2022). Indeed, Bhardwaj et al. (2022) found that organisational failures contributed to 80% of the accidents’ root causes and concluded that while human activities are involved in most accidental events, they are mainly motivated by organisational errors. More recent, but still very few studies, are capitalising on advanced data analytical techniques. For instance, Wang et al. (2021) use text mining and data analysis techniques to highlight the role of working conditions as accident risk indicators.

Clearly, from this review, focusing mainly on human factors and errors, in particular, some other risk indicators related to the broader and inter-related ecosystem within which any maritime vessel operates and exists, might be being overlooked. Generally, causes of accidents are not considered at a level beyond that of the ship, with a few rare exceptions. Some examples confirming these exceptions are the deficiencies of Port State Control (PSC) (Fan et al., 2018; Wang et al. 2021); violation of regulations (but it is unclear whether these were due to the decision-making of the Captain and/or shipowner) (Shi et al. 2021); insufficient government supervision of ship owners and shipping companies (Wang and Yin 2020). Thus, there appears to be a gap in our knowledge and understanding of the causes of accidents that largely neglect the role of the broader ship ecosystems (communication with other ships, communication with the shore, maintenance and investment choices made by the shipowner, managerial choices such as the number and level of qualification of the crew, manning and navigation decisions made by the shipowner/port agents to the ship) as risk factors. This paper aims to address this gap.

### Maritime accident investigation reports: an overview

In addressing the research objectives, this study used the publicly available maritime accident reports submitted to the IMO from 2011 to 2021 as the primary data source. However, very few studies have used accident reports because there is a fundamental lack of standardisation of information in the largely qualitative reports, which makes them difficult to analyse (Fan et al. 2020; Zhang et al. 2019a, b). Thus, there is a need for the data to be prepared more systematically with more consistent definitions so that they can be used to investigate socio-technical causation factors in accident modelling (Mazaheri et al. (2015). However, this is very time-consuming, and of the few studies that use accident reports, they either only use a limited number of reports as their database or the analysis is too tentative (Fan et al. 2020; Zhang et al. 2019a, b).

This study addresses these shortcomings by demonstrating how such data can be effectively analysed using novel text-mining techniques to extract new insights into maritime accident risk factors. In a maritime accident, each respective national reporting Administration must submit marine casualties and incidents reports and complete marine safety investigations to the International Maritime Organization (IMO 2021a, b). According to the Code of International Standards and Recommended Practices for a Safety Investigation into Marine Casualty or Incident, this includes information related to vessel details, other vessels involved, occurrence type including external factors (weather and light), voyage data, consequences, injuries and fatalities, description of occurrence (including sequence of events) and recommendations for preventing the accidents in future (IMO 2021a). In all cases, marine accident investigations specifically focus on “*fact finding rather than guilt finding*” (Garrett and Teizer 2009: 756). As many of the filed reports state, they are intended “*to promote the safety of life and property at sea and to promote the prevention of pollution*” and are not intended to apportion blame or determine liability (RMIMA 2020).

Under the Safety of Life at Sea (SOLAS) regulations I/21 and XI-1/6, and the MARine POLlution (MARPOL) international convention, articles 8 and 12, the IMO requires respective nation-states to submit accident casualty reports to their Global Integrated Shipping Information System (GISIS) (IMO 2014, 2021a, b). In order to try to rationalise and consolidate the international reporting of maritime accidents, the IMO has worked with the European Maritime Safety Agency (EMSA) to harmonise reporting procedures with the European Maritime Casualty Information Platform (EMCIP) and avoid duplication. The GISIS module is organised into five sections or appendices (IMO 2014), where data can be entered into pre-set fields. Completion of the first two appendices is required as a minimum and include Appendix (1) generic information related to the accident, including the State, numbers of ships involved, generic casualty data and recommendations to prevent future marine casualties; Appendix (2) factual information related to each ship involved in the incident/accident including ship particulars, voyage data and casualty data.

For the remaining appendices, the IMO requests that the Administrators provide as much data as possible. The data fields for Appendix (3) provide the input of casualty analysis data related to each ship involved, including accidental events and contributing factors. Appendix (4) enables the input of supplementary information according to the particular circumstances relating to the respective casualty/incident. Appendix (5) of the IMO GISIS has 30 field value options that cover the details of the accident and are based on the EMSA’s Casualty Analysis Methodology for Maritime Operations (CASMET) approach (Cardis 1999), which takes a human-centric perspective of maritime accident analysis. This methodology for classifying human and organisational errors aims to enable meaningful comparison between countries and develop a common taxonomy for storing information related to marine casualties. Thus, the IMO (GISIS) data fields include the respective marine administration, investigation and nationality; safety recommendation focus; location, casualty event and severity; external conditions (sea, wind, visibility, weather); ship or task operation (what it was doing); goods on board (including hazardous materials); accident event details; details of the crew; training levels on board; error type; human contributing factors (temporary such as fatigue and permanent such as functional impairment); operational contributing factors (including social environment, supervision, workplace condition, emergency response); management/organisational contributing factors (such as operations, safety, organisation and general management, personnel); equipment, and external agencies (including systems and tasks).

Based on these observations, we contend that current causation analyses focus on a too narrow scope of factors limited to issues related to the ship and those individuals on board. The accident investigation auditing checklists (as recommended by maritime institutions and organisations) tend to focus mainly on the level of the individual and, to a much lesser extent, on organisational factors. One of the consequences of this approach to accident investigations is characterised as conforming to the What-You-Look-For-Is-What-You-Find (WYLFIWYF) principle (Hollnagel 2008; Lundberg et al. 2009). However, as Besnard and Hollnagel (2014) assert, accident investigation is a social process where causes are constructed rather than found, where the purpose is not to find causes but to build explanations. Thus, to broaden our understanding and improve maritime safety, we need to enlarge the scope of analysis from an individual human-centric level to a much larger maritime ecosystem of actors.

### Accident investigation report analysis: an overview of methods and techniques in the literature

In order to broaden the scope of analysis, the methodologies and techniques for analysing accident investigation reports by scholars in a range of different industrial contexts was first reviewed. From this review, two main approaches—statistical and text mining—emerged as most apt for analysing maritime accident reports.

#### Statistical analyses

The combination of descriptive and inferential statistics (in particular ANOVA—Analysis of Variance) is commonly used by scholars investigating causes of accidents and fatalities in different industry contexts, for instance, in road and maritime transportation, aviation and construction. The following provides examples of how ANOVA has been used to inform accident research in these different contexts.

In a study of road traffic accidents and fatalities, the data from the United Kingdom’s (UK) Road Transport Department was analysed using a combination of descriptive and inferential statistics (ANOVA) and machine learning techniques to better understand the factors that impact the number of accidents and associated fatalities (Haynes et al. 2019). In the aviation sector, where 60% of accidents are attributed to pilot error, Wang et al. (2018) used inferential statistics (e.g., ANOVA) to find significant differences between pilots’ flare operations and types of abnormal landing events (hard and long), which increase the risk of accidents. Again ANOVA was used in a construction industry study (Choe et al. 2020) to reveal that there was no significant difference in safety practices between contractors and sub-contractors (inter-organisationally) but that there was a difference within organisations (intra-organisationally) between safety practices set by head office and the safety capabilities at the construction site.

In the maritime transport sector, Yim (2017) analysed 1,606 accident reports from the Korean Maritime Safety Tribunal to determine the impact of seafarers’ shortcomings on the types of accidents. They classified these ‘deficient abilities’ or human error as Skills (lack of experience), Rules (non-compliance) and Knowledge (lack of knowledge). Using ANOVA statistical tests and cumulative frequencies, they found that skill, rules, and knowledge impacted both accident type and vessel type and that these three factors are inter-related. They concluded that seafarers’ deficiencies in skills, rules and knowledge inherent in maritime accidents could be identified in 70% of the cases using these methods but that there was a need to develop more accurate methods for analysing maritime risk factors in the future.

#### Text mining techniques

Text mining techniques continue to advance rapidly and are increasingly being used in the context of transport safety to identify accident characteristics and address them in a range of transport sectors, including rail, road, air and sea.

In his study Brown (2016) used text mining techniques and types of ensemble modelling (or clusterisation) to understand better the characteristics of accidents and the contributory factors of railway accidents in more depth. By ‘mining’ the narrative reports of rail accidents, he used a combination of analytical methods to identify accidents of interest and highlight any relationships between contributors to accidents. This study demonstrated the value of qualitative narratives in accident reports and how text mining can improve understanding of risk factors of accidents and their severity. In the construction industry context, Zhang et al. (2019a) used text mining and natural language processing techniques to classify the causes of accidents based on the types of dangerous objects and the risks they pose in the workplace.

These studies highlighted how text mining analytics techniques enable deriving information processes not previously known from narratives of accidents in different transport modes. Here, these techniques are adapted to the context of maritime accident reports to address the research objectives of this study and will be explained in more detail in the following sections.

#### Summary of literature findings: contradictions and inconsistencies

In their study, Baniela and Rios (2011), drawing on 256 maritime accidents worldwide, found differences between the seriousness of accidents based on age (the older, the more serious), size (the smaller, the more dangerous) and classification of the vessel. Similarly, Li et al. (2014), drawing on a sample of 8023 maritime casualty reports drawn from a combination of Lloyds’ Register of Ships, World Shipping Encyclopedia and the IMO, found that vessel safety was primarily influenced by a vessel’s age (the older, the safer), size (the larger, the less safe), type (cargo and passenger are riskier), classification society and flag states. Even within these ostensibly similar headline findings, there are contradictions as both studies find a link between vessel safety and vessel size. However, how these impact accidents are in direct opposition to each other: older vessels were found to be associated with both more (Baniela and Rios 2011) and less (Li et al. 2014) safety and smaller vessels are found to be less safe (Baniela and Rios 2011), and larger vessels are found to be safer (Li et al. 2014). More recently, Bye and Aalberg (2018), examining navigation accidents in Norway, highlighted the contradictions of their own and other studies into causes of accidents, where some are associated with visibility, speed, and flag states but in opposing ways (good vs poor visibility; high vs low speed and white vs grey/black flag registration).

On the other hand, Fan et al. 2020, developed and tested a model built on the data from IMO maritime accident reports using Bayesian Networks and Tree Augmented Networks, which provides more nuance by presenting probabilistic outcomes of the interaction of human risk factors with vessel characteristics that are most likely to result in maritime accidents. Their findings revealed that the critical risk factors for all accident types (in descending order of importance) are ship age, ship operation (e.g. towing, piloting, at anchor, fishing, on passage etc.), voyage segment (its location, for instance, in port, mid-water, in transit), information (reliability, validity, and currency of the information provided) and vessel condition (which includes size, the complexity of propulsion mechanisms and any modifications) and that there is an interdependency between these risk influencing factors. However, their model differentiates between the vital factors contributing to different types of accidents and how different states of single variables impact the probability of a particular accident type occurring.

## Methodology

**Fig. 1:**
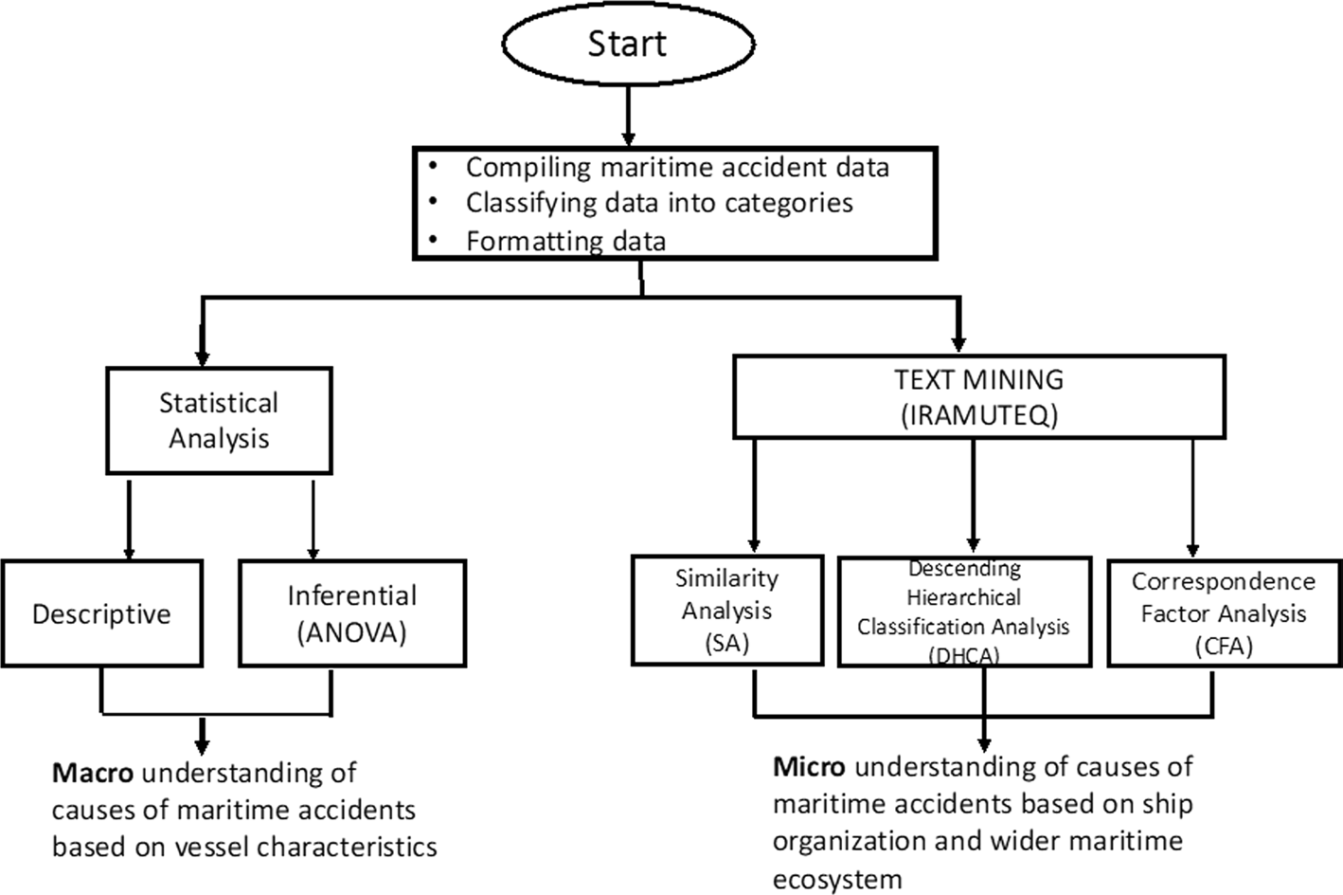
Summary of research methods applied. Source: Authors

Maritime investigation accident reports submitted to the IMO are a valuable primary qualitative data source containing detail including navigational circumstances, actions taken, the chain of events, and environmental conditions, as already detailed earlier. The research methods and steps followed in this study are summarised in Fig. 1. First, data must be prepared by compiling, classifying and formatting it. Once the data was prepared, statistical and text mining methods using the IRAMUTEQ software were applied and explained in detail in the next sections.

### Statistical analyses

#### Database set-up and descriptive statistics:

The data for this research was generated from 504 qualitative maritime accident reports from the IMO database covering 2011–2021. The selection and data generation protocol involved the following steps:

1. Searching the online IMO database for accident reports falling within a ten-year period from 12 July 2011 to 12 July 2021,
2. Manually screening each of the reports to ensure they included all the information required for this study (namely checking for the presence of vessel and accident details, the seriousness of accidents including casualties and accident narratives),
3. Systematically quantifying and organising the data to make it suitable for applying the relevant statistical and text mining techniques. In line with the published research into maritime accident risk indicators, we set up the databases with the four most commonly used variables. Table 1 details the eighteen items in four categorical variables extracted from the dataset.
4. Once the variables were set-up, the corpus was formatted into quantitative variables for statistical analysis and free text for content analysis. This process followed the IRAMUTEQ protocol (see "Database set-up and descriptive statistics" section), where each accident report from the IMO database was uploaded into our local database. We concatenated all the reports into one corpus, separating each report with 4* followed by each of the four variables with the corresponding modalities (description of vessel characteristics and context as described in Table 1). Below we present an example of the Costa Concordia accident for illustration:

**Table 1:** Vessel characteristics variables and description. Source: Authors based on the Extracted IMO Accident Reports (2011–2020)

Independent variables	Description of vessels characteristics	No. of vessels involved in accidents	Source
Flag state	White (e.g., Denmark, Marshall Islands, Bermuda, Singapore, Japan, Portugal, Panama, US) Grey (e.g., India, Switzerland, Ukraine, Philippines, Lebanon, Poland) Black (e.g.Cameroon, Albania, Belize, Moldova) Other	441 22 19 22	Paris MoU Vessel Flag Classification (2021–2022) based on inspection of the vessel’s risk profile, international compliance with safety and pollution prevention measures
Age	Old (A_O): > 20 years Middle (A_M): > 10- < = 20 years Young (A_Y): < = 10 years Other (A_Oth)	122 147 232 3	Fan et al. (2018)
Vessel type	Bulker (VT_B) Container (VT_C) General cargo ship (VT_GCS) Tanker (VT_T) Passenger (VT_P) Offshore (VT_O) Other (VT_Oth)	157 71 116 75 46 3 36	
Size	Large (S_B) > = 20KGT Medium (S_M) > 2KGT < 20KGT Small (S_S) < = 2KGT	264 182 58	
Dependent variable			
Marine casualty	Number of deaths	502	

^*^Total number of injuries = 744. **Total number of IMO accident reports = 504 KGT = thousand gross tonnage

****[indicates new report] *VN_COSTA_CONCORDIA [name of vessel] *F_F_W [flag state] *A_A_Y [age of vessel] *MC_MC_VS [marine casualties] *S_S_B [size of vessel] *AccDT_13-01-2012At21h00 [date and time of accident] *VT_VT_P [vessel type]

#### Analysis of variance (ANOVA)

An ANOVA was applied to the data to examine whether certain ship characteristics were associated with the more serious outcomes of accidents (i.e., deaths). Here, the analysis of variance looks for differences in the type of casualty (number of deaths = dependent variable) between the groups with ship characteristics (independent variables), namely type of ship, age of the ship, size of the ship, flag state or country in which the ship is registered, to determine whether certain characteristics are related to more serious accident outcomes (number of deaths) than others. In order to conduct the ANOVA, three main assumptions had to be met, so we tested the data for normality, equal variances and independence.

### Text mining analysis (IRAMUTEQ)

The major aim of text mining is to find patterns in unstructured and semi-structured text, including some but limited metadata such as dates, titles, names etc. (Brown 2016). Through pattern discovery, the contents of largely unstructured/semi-structured qualitative documents (such as the accident reports in this study) can be characterised to address research objectives. Here, text mining provides much richer information and insights into accident characteristics not available from only fixed field entries, which are often limited to the schema and structure of the researchers’ objectives and dataset (Brown 2016).

For this analysis, IRAMUTEQ (Interface de R pour les Analyses Multidimensionnelles de Textes et de Questionnaires) created by Pierre Ratinaud was used. IRAMUTEQ is a free open source text mining software based on the statistical package R and Python, that has a ‘user-friendly interface’ and ‘provides quality graphical output’ (Sarrica et al. 2016). Until recently, this software could only accommodate the French language but has since included dictionaries in several languages, including English and Portuguese (Souza et al. 2018), and is now being used more frequently for qualitative research in social sciences, psychology, and health studies to analyse large textual corpora to highlight thematic categories and analyse similarities (Chaves et al. 2017; Sarrica et al. 2016).

#### Preparation of the corpus

Firstly, to prepare the text mining corpus, the IRAMUTEQ instructions were followed, and the same variables as those used for the ANOVA were included. Then, pre-processing and feature extraction steps were taken, including transforming all text to lower case, removing punctuation to reduce the training data size, and removing stopwords, which are common words of little value and are context-specific (Zhang et al. 2019a).

#### Text mining analyses

As per Fig. 1, three techniques available in the IRAMUTEQ software were used to analyse the data:

1. *Similarity analysis (SA)* based on the graph theory, identifies the co-occurrences of words, providing information about their connectivity to help identify the structure of the context of a corpus of text. It also identifies the shared aspects and specificities of the descriptive variables identified in the analysis (Marchand and Ratinaud 2012). Analysing the proximity of the most frequent terms (in this study, 143 most frequent terms) presents the ‘universes of words’ (Fig. 3), constituting the central terms and how they relate to other terms by proximity. This study used the co-occurrences index, which produces similar results to the ChiSquare index).
2. *Descending Hierarchical Classification Analysis (DHCA)* was used to drill down into the main themes developed in the corpus using the Reinert Method (Fig. 4a), which is a dendrogram that provides an overview of the twenty words that are most correlated to each cluster (highest chi-squared),. Then they were contextualised to show how these words were used by highlighting extracts in the reports to interpret each cluster’s content and relate it to the role of different actors that intervened throughout the maritime ecosystem concerning the accidents.
3. *Correspondence Factor Analysis (CFA)* was used to produce a graphical visualisation (Fig. 4b) of the former DHCA “*of the proximities, oppositions and tendencies of the text segments (TS) or corpora classes; locating these elements in a Cartesian graph with factors generated from their classifications and allowing graphical visualisation of the co-occurrence between words and the possible communities in which they coalesce*”(Bienemann et al. 2020:3–4)

## Findings

### Statistical analyses: the macro level of understanding of maritime accidents

#### Descriptive statistics

In the dataset of IMO reports extracted for this study, there were 504 maritime accidents over the decade from January 2011 to December 2020 and 502 deaths and 744 injuries (Table 1). The average number of reported deaths for the period was nearly 1 (0.996). The majority of the vessels involved in accidents resulting in serious casualties (deaths) were large (over 20,000 gross tonnage), operating under a white flag state, young (under ten years old) and bulker vessels. More descriptive details are summarised in Table 1.

**Fig. 2:**
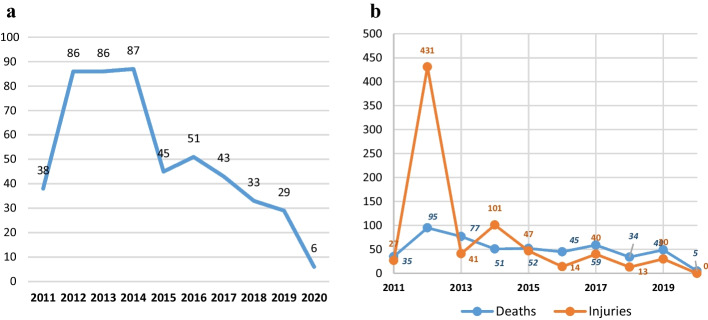
**a** Total annual number of maritime accidents (2011–2020). Source: Based on IMO dataset. **b** Total annual number of deaths and injuries in maritime accidents (2011–2020). Source: Based on IMO dataset

The total number of maritime accidents has been falling over the past decade, as illustrated in Fig. 2a. However, since that time, the reason for the sudden halving of accidents in 2015 and a more steady fall in accident numbers is unclear. It will be interesting to monitor this figure over the next few years to see whether this is a consistent trend that marks a real improvement in maritime safety.

The annual numbers of casualties (deaths and injuries) over time are summarised in Fig. 2b and tend to fluctuate. The two extraordinary peaks of casualties in 2012 and 2014 resulted from very serious single accidents (for example, in 2012, the Rabaul Queen led to 146 deaths and 247 casualties; the Costa Concordia led to 32 deaths and 152 casualties and Doola n°3 led to 11 deaths).

The 502 accidents reported involved 501 maritime vessels; out of the 502 accidents reported, no single vessel reported more than two accidents over the 10 years. Of the three vessels reporting two accidents in the space of a decade, there were four deaths, four injuries, and no more than 1 or 3 injuries in a single accident. The average time between the accidents on the same vessels was 24.6 months, with the minimum period being 13 months and the maximum being 44 months. Two of the three vessels with repeated accidents were passenger ships, and the other was a container/bulk carrier ship. The figures in 2020 approaching 0 were due to the lockdown and circumstances of the economic slowdown resulting from the COVID-19 pandemic.

#### Analysis of variance (ANOVA) results

The variables summarised in Table 1 were used for the analysis of variance. The metric for accident seriousness was the number of deaths, which was set as the dependent variable. The other categorical variables, Flag State, Vessel type, Age and Size, which are characteristics of the maritime vessel, were set as independent variables to examine the differences between these categories.

In this case, the assumption of normality was tested, and the distribution of the data in the dataset (number of deaths) was not normal, which is not unusual in the social sciences and reflects the underlying nature of the construct being measured—namely characteristics of marine vessels and number of deaths. Consequently, we used the non-parametric Kruskal-Wallis test, which does not require the assumption of normality or equal variance. In addition, the final assumption of independence is also fulfilled as the observations in each group (accident report) are independent of the observations in any other group and were obtained by random sampling (Pallant, 2013). Based on these assumptions, a one-way ANOVA test was used to determine whether there was a difference in the number of deaths reported (dependent variable) based on the different characteristics of marine vessels (independent variables). The ANOVA revealed no significant difference in the number of deaths across the different groups of Flag State and vessel type (Table 2). However, the ANOVA results found significant differences in vessel age and size.

**Table 2:** Summary of ANOVA results. Source: The Authors

Kruskal Wallis test (ANOVA)	Chi-square	Df	*p*-value
Nbdeaths by FlagState	5.4424	3	0.1421
Nbdeaths by VesselType	11.063	6	0.08646
Nbdeaths by Age	11.552	3	0.009085*
Nbdeaths by Size	8.9828	2	0.0112*

^*^significant *p* < 0.05

In order to explore these findings further, the mean ranks for the two significant characteristics were examined and summarised in Table 3. First, Number of Deaths versus Age: there is a significant difference in the number of deaths across the different ages of vessels (*p* = 0.009). An inspection of the mean ranks for the age group suggests that in this case, most deaths were reported on older vessels 20 years or over, followed by young vessels ten years or less, with the least number of deaths reported on medium-aged vessels between 10 and 20 years old. Regarding the size, there is a significant difference in the number of deaths across the different sizes of vessels (*p* = 0.01). An inspection of the mean ranks for the vessel size group suggests that the small-sized vessels (less than 2KGT) reported the highest number of deaths, followed by large vessels (more than 20KGT), with medium-sized vessels (between 2 and 20KGT) reporting the least number of deaths.

**Table 3:** Summary of mean ranks for significant vessel characteristics and number of deaths. Source: The authors

	*Significant vessel characteristic	Mean ranks
Number of deaths (Nbdeaths)	Vessel age: A_O (Old) > = 20 yrs A_Y (Young) < = 10yrs A_M (Medium) > 10 < 20yrs	2.132231 1.206897 0.639456
	Vessel size: S_S (Small) < = 2KGT S_B (Big) > = 20KGT S_M (Medium) > 2KGT < 20KGT	3.66667 1.022727 0.846154

The results from this part of the study are consistent with the literature in that they exhibit inconsistencies when compared with the findings from other studies investigating maritime characteristics as risk factors in maritime accidents. This will be discussed in more detail later.

### Text mining techniques

#### IRAMUTEQ analysis of similarities

**Fig. 3:**
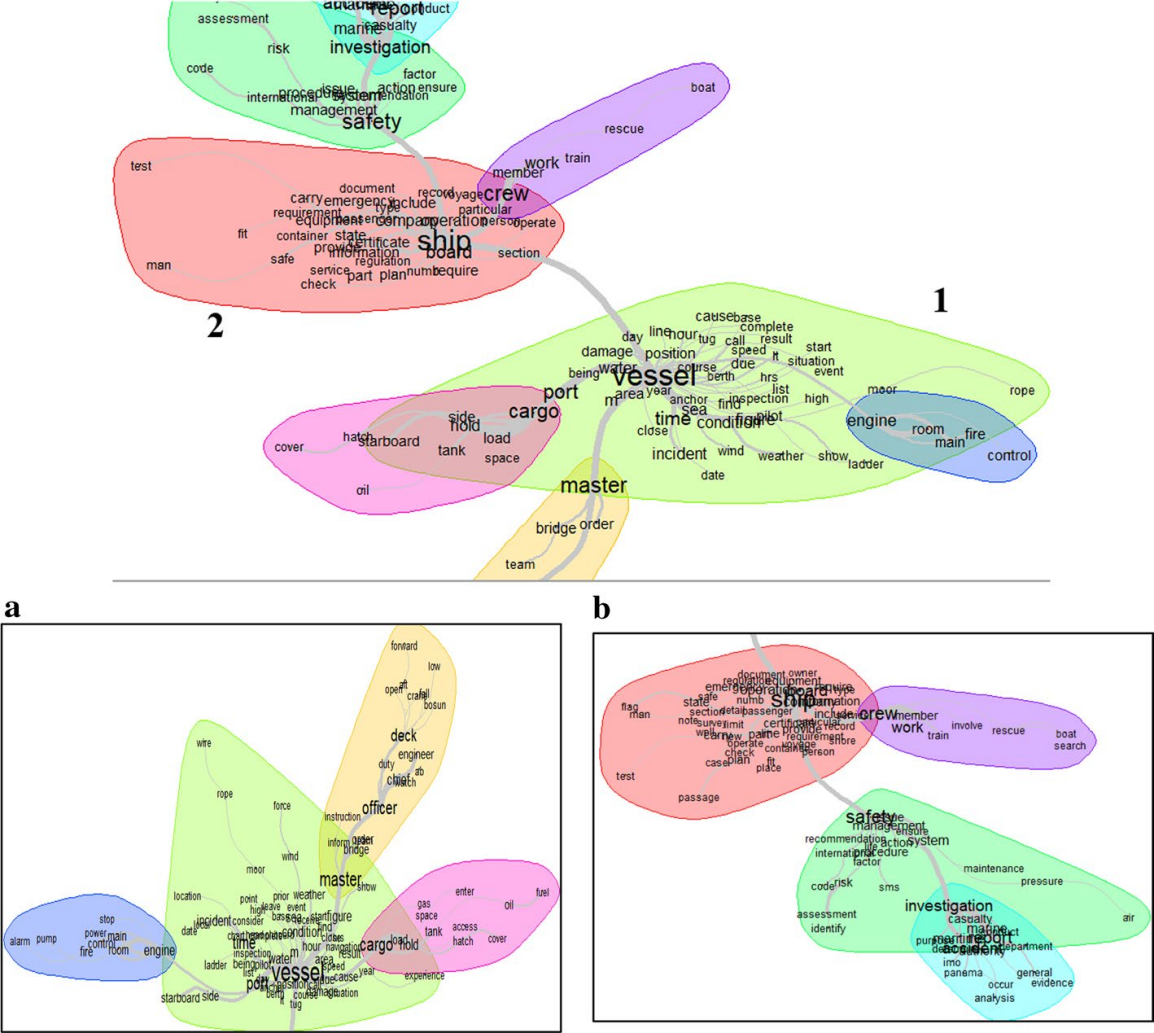
IRAMUTEQ analysis of Similarities of IMO dataset (based on co-occurrences of 143 most frequent words). Source: The authors. **a** Universe of words around the term “vessel” from the IRAMUTEQ Analysis of similarities in IMO dataset. **b** Universe of words around the term “ship” from the IRAMUTEQ Analysis of similarities in IMO dataset

Firstly, an analysis of similarities with the most frequent 143 terms and their universe of words was developed. Figure 3 shows that all maritime reports are structured around two main ‘universes of words’: (1) “vessel” incorporates explanations of the context and causes of accidents related to technical issues and the ship’s physical structure; (2) ‘ship’ incorporates explanations of the context and causes of accidents related to the ship considered as an organisation and includes the role of individuals.

Examining the two main universes of words in more detail, the first, ‘vessel’, reveals four main universes of words presented in Fig. 3a and detailed as:

1. *vessel* (central in green), related to general navigational information (e.g., location, date, starboard, moor), external factors (e.g. weather, wind), technical issues regarding equipment (e.g. ropes, wire), and provides individual errors related to the use of material (e.g.ladders, tugs).
2. *engine* (left in blue), related to technical explanations of the accident (e.g. gas, space, tank, oil, fuel, alarm, pump, engine, deck).
3. *cargo* (bottom right in pink), related to leaks of fuel or oil, and explosions related to gas.
4. *master* (top right in yellow), mentions the roles and actions of the actors (master, officers, bosun) within the engine room or the deck concerning the accident.

Focusing on the second universe of words, ‘ship’, also reveals four main universes of words presented in Fig. 3b and detailed as:

1. *ship* (central in pink) describes actions taken by individual crew members (with verbs such as check, fit, test, include, and operate). The shipowner is also mentioned.
2. *the crew* (top right side in purple) comprises fewer words than the three other universes, but the internal ship organisation is mentioned in the description of the tasks related to work and the search and rescue operations.
3. *safety* (bottom right in green) is dedicated to the ship’s compliance with international procedures (ensure, code, management, procedure, assessment).
4. *investigation* (bottom right in blue) including aspects of the procedure and evidence of accident causes.

Overall, the main themes that are covered in maritime accident reports are mainly developed through the lens of technical issues due to the physical structure of the vessel or from ships seen as organisations (tasks and management of the crew)n including individuals (errors due to individuals, problems of behaviour. From this analysis, apart from the technical issues, the risk indicators of accidents focus mainly on the on board ship organisation and individual perspective, with very little information on the broader role of the actors beyond the limited boundaries of the ship.

#### IRAMUTEQ clusterisation analysis

**Fig. 4:**
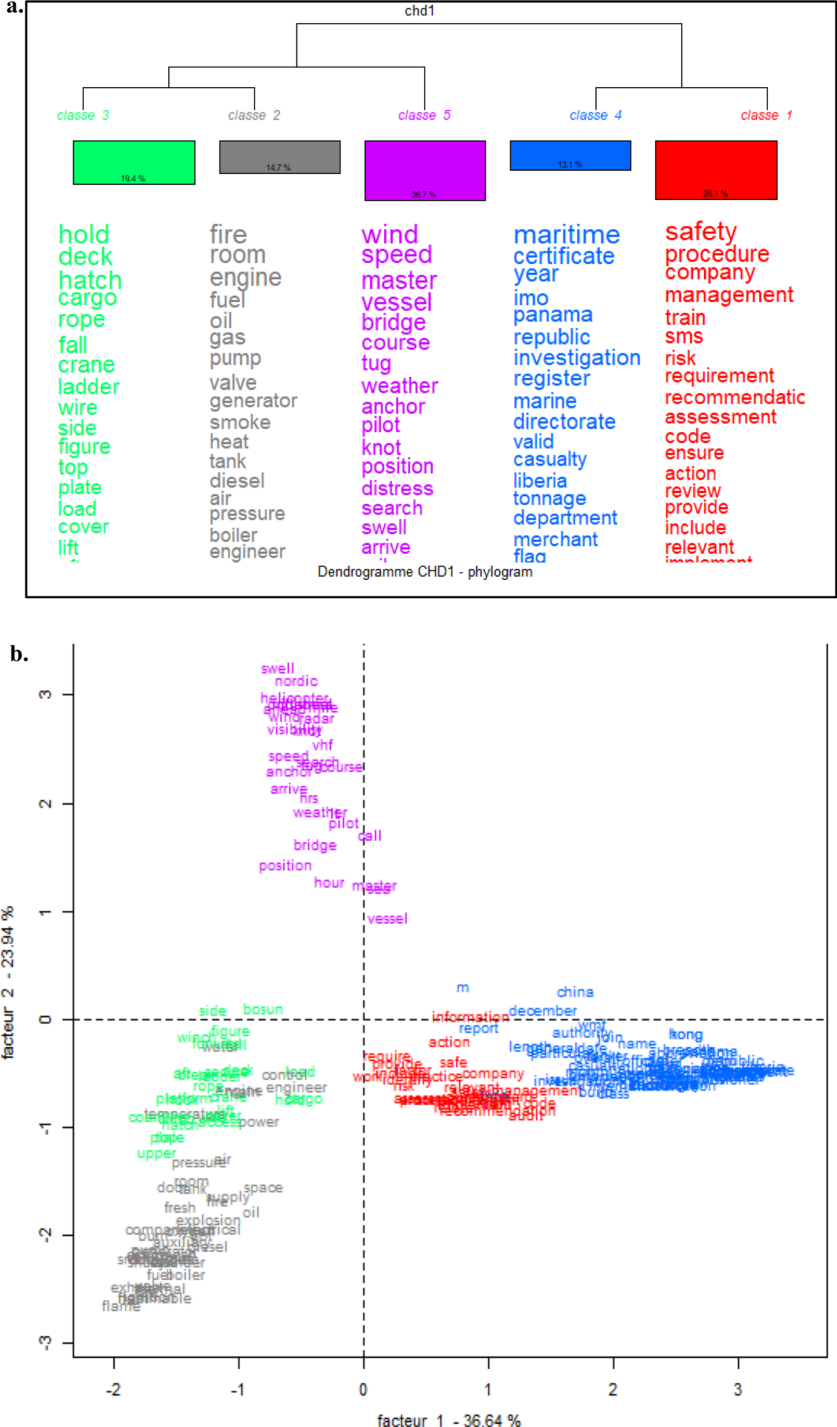
**a** Dendogram of clusters* and associated word extracts (DHC) in IMO dataset. *Cluster is reported as classe in IRAMUTEQ dendogramme). Source: The authors. **b** Graph of correspondence factor analysis (CFA) of IMO dataset. Source: The authors

By applying the clusterisation technique to the IMO dataset, five clear clusters emerged. There are presented in the dendrogram in Fig. 4a.

1. Cluster 1 (red) deals with the safety procedures and their management within the ship organisations (‘procedure’, ‘safety’, ‘company’, ‘train’) and represents 26.14% of total text segments (TS).
2. Cluster 2 (grey) deals with accident narration on board related to equipment failures (‘fire’, ‘room’, ‘engine’) and represents 14.68% of total TS.
3. Cluster 3 (green) deals with accident narration on board directly caused by individuals on board (‘hold’, ‘hatch”, ‘deck’, ‘fall’, ‘ladder’) and represents 19.38% of total TS
4. Cluster 4 (blue) deals with international regulations and safety rules that are checked during the investigation (‘certificate’, ‘imo’, ‘panama’) and represents 13.12% of total TS.
5. Cluster 5 (purple) describes external factors during navigation (‘wind’, high ‘speed’ of the vessel or ships close to the vessel in case of collision, bad weather, poor visibility) and represents 26.7% of total TS.

The results of the correspondence factor analysis (CFA) in Fig. 4b indicate that Cluster 1 and Cluster 4 are linked as they represent the international regulation of the ship with issues related to compliance and/or behaviour from the ship or the ship’s broader external ecosystem (flag state, authorities, merchant shipping directorates, classification societies). These two clusters highlight the responsibilities of external actors related to the ship.

Similarly, Clusters 2 and 3 are also linked as they both describe the circumstances of the accident. Cluster 2 is for technical matters and Cluster 3 is for human errors. These two clusters are also, to a certain extent, linked to Cluster 5, which completes the description of the accident by describing the external factors relevant to navigation (e.g. weather, speed of the ship). In summary, the CFA has revealed the text segments (TS) related to regulations and their applications on board (Cluster 1 and 4), and narration of the accidents with a different focus (technical issues in Cluster 2), errors directly attributed to individuals (human errors in Cluster 3) and external factors (mainly weather and speed in Cluster 5).

##### Analysis of cluster content

**Fig. 5:**
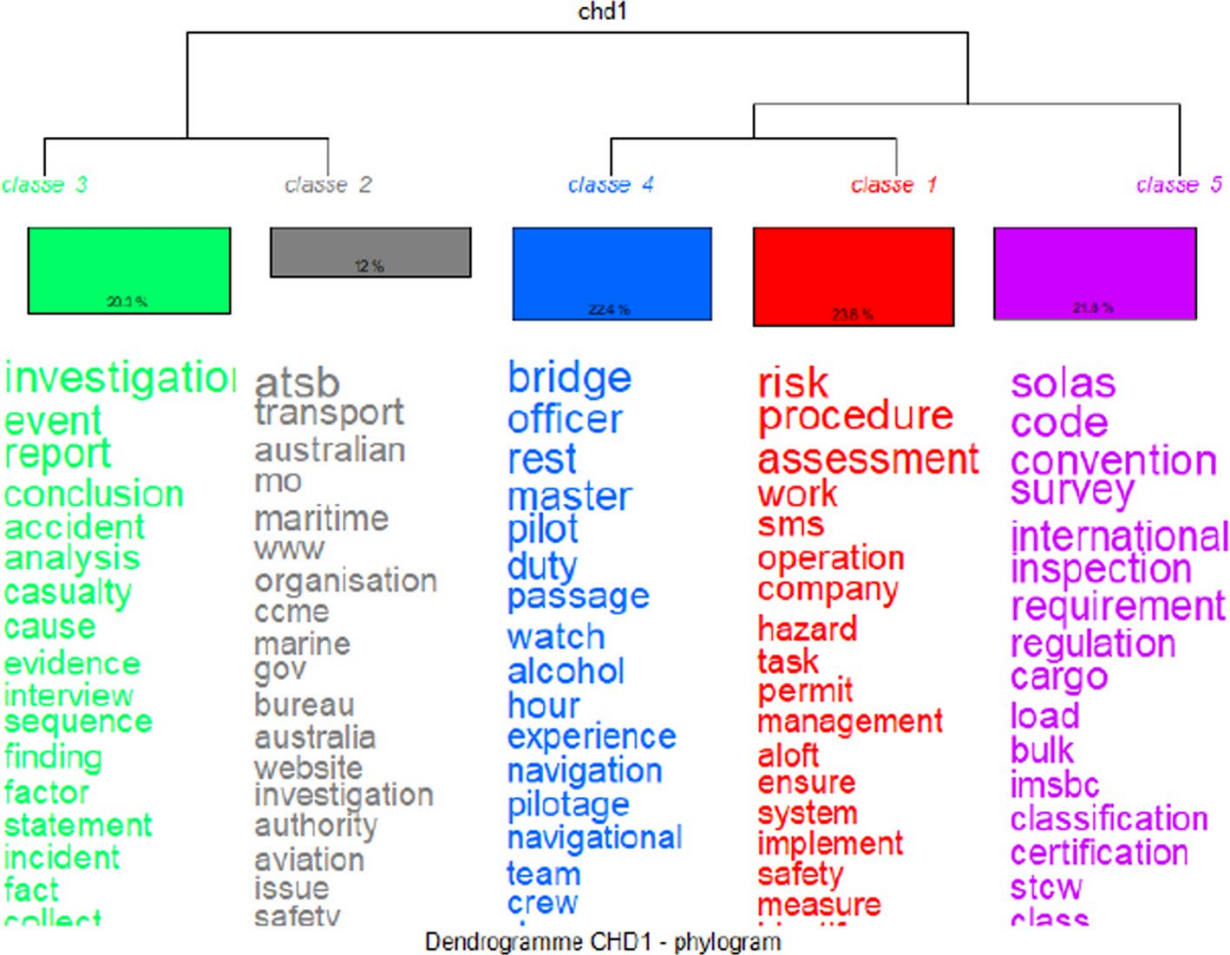
Dendrogram of clusters extracted from the sub-corpus of cluster 1 and associated word extracts (DHC) in IMO dataset. (* Cluster is reported as Classe in IRAMUTEQ dendrogram). Source: The authors

**Fig. 6:**
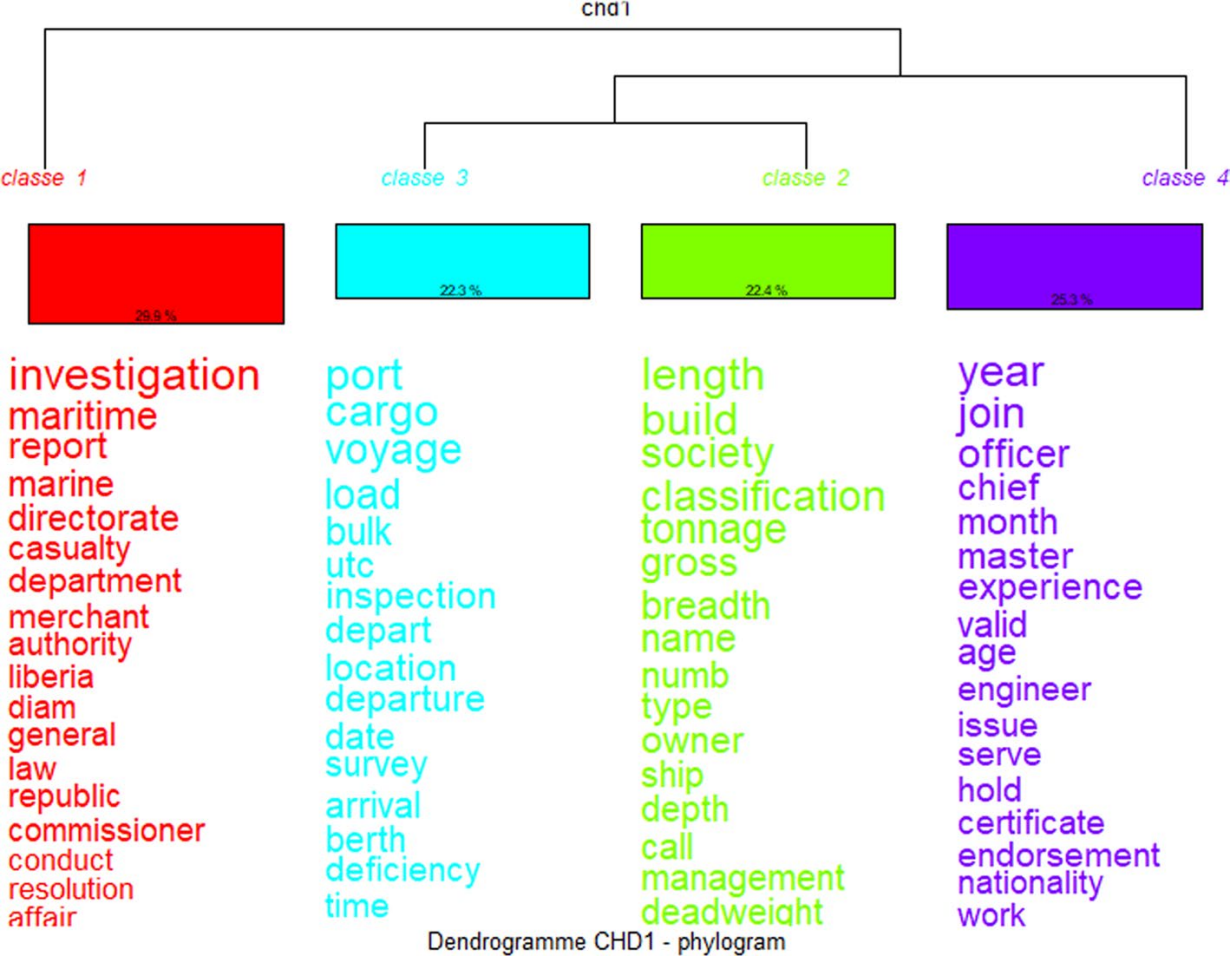
Dendrogram of clusters extracted from the sub-corpus of cluster 4 and associated word extracts (DHC) in IMO dataset. (* Cluster is reported as Classe in IRAMUTEQ dendrogram). Source: The authors

Having established the clusters highlighted in the dendrograms (Figs. 5 and 6) and the linkages between them, the next phase is to examine each cluster’s content in more depth. Here, the extracts’ details associated with each cluster will be examined regarding their contextual use of the words most correlated with its cluster. Again, quotations from accident reports will be used, but the details of the vessels are abbreviated to ensure some anonymity. This will provide an understanding of the causes and the corresponding maritime entity/actor (e.g., individual, crew, shipowner) that might be responsible for them.

**Fig. 7:**
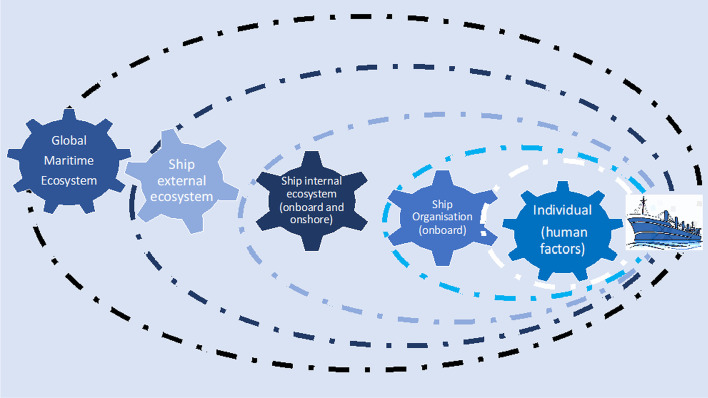
Accident maritime ecosystem (AME) framework. Source: Authors

Cluster 1 reveals three main sub-themes with two corresponding responsible entities/actors—the crew and the shipowner. Here, the accident reports generate sub-theme (i) such as violation of safety procedures, (ii) lack of skills regarding procedures (“*the crew is completely ignorant of safety measures*” VN_D), or (iii) an insufficient safety culture (“*safety culture is not implemented and crew motivations toward safety and security found very limited poor*”—VN_A), which reveals some dysfunctions at the level of the crew. Within this cluster, the responsibility of the shipowner is also reported (but less frequently). Several problems are highlighted, the most frequent of which is the lack of maintenance on ships (“*the absence of investment in the maintenance of sea breeze along with the lack of planned surveys that were….. there was no hull and machinery insurance (..) a loss of contact between the owners and managers*”*-* VN_S_B). We consider this to constitute the foundation for what we will term in Fig. 7 the ship’s internal ecosystem. Creating a sub-corpus with Cluster 1 enabled us to understand its content further.

Clusters 2 and 3 are dedicated to the investigation procedure concerning the authorities. Conversely, Cluster 1 is related to the shipowner’s decisions and management (mentioned as “the company” in most reports), which relates to Cluster 4 depicting the decision of the actors leading the management on board (‘the officers’, ‘the master’) regarding the command of the team on the bridge in coherence with international conventions. In a nutshell, the sub-corpus analysis of the main Cluster 1 leads to two sub-main sub-themes. On the one hand, we have leadership and management on board; on the other hand, the shipowner’s decisions, and their consequences on board.

Cluster 2 provides information on three main technical issues related to the vessel: (i) deficiencies in flow pressures of engines are frequent; (ii) other types of deficiencies are mentioned concerning the structure or material on board; (iii) several reports describe the destruction of the structure/equipment of the ship following a fire. This cluster is very descriptive and does not link these technical issues to any specific actors. Nevertheless, we consider this to constitute the foundation for what we will term in Fig. 7, the ship’s organisation as the issues relate to the ship.

Cluster 3 points out the *behaviour of individuals*, and to a lesser extent, the crew’s behaviour, to explain several causes of the accidents. Firstly, non-compliance of procedures from individuals (“*the bosun was not wearing a safety harness*” VN_PM; “*there was no safety checklist developed to access the poop deck, and it was not declared a restricted area*” VN_L). Other explanations are related to a lack of maintenance of the material on board (“*the outer surfaces of the wire rope were mostly covered in heavy grease (…) several areas of the rope were drier and appeared rusted*” VN_NB). In addition, several errors are directly and most frequently attributed to individuals (“*he apparently wasn’t aware that the hatch over the cargo was not completely closed while walking backwards still guiding the grab he fell into the cargo hold*”, VN_SW). Finally, a few human behaviours are mentioned but without being able to provide any explanations (“*there is little known about the reasons why an experienced deck officer acted as he did (..) he did not call either the master or the chief officer when the cargo slops discharge was completed*”, VN_H). All in all, both clusters 2 and 3 focus on the causes that are described on board. We consider this to constitute the foundation for what we will term in Fig. 7, the individual.

Cluster 4 sheds light on several actors’ roles beyond the ship’s narrow boundaries. Among them, flag administrations are criticised for extending patents for vessels that should not be allowed and ships being grounded without first informing the flag administration with no reaction from the authorities (“*no action was taken against the vessel from the authorities when she went grounded in 2010 (…) the flag administration was not officially informed by the authorities*”, VN_LM). Moreover, classification companies and merchant shipping are sometimes mentioned as actors who authorise ships to continue on their route even though the ship’s general quality standard has been questioned (“*following the completion of the temporary repairs required by the classification society TT, the flag state approved the insurance for short term certificates so that the ship could proceed to Dubai..*”, VN_R_D). Other times several classification societies are contracted for different certificates related to the same ship (“*neither the master nor the owner advised class of any defects (…) the ship had been classified by the society BB whereas the port security certificates were allowed by DD*”, VN_S_B). Finally, all these stakeholders appear to have complex inter-relations, notably in terms of externalisation of tasks and related responsibilities from public to private organisations (“*this organisation has been assessed by the flag state and has the delegation of authorities to perform statutory certifications and services on behalf of the flag state (…) The international association of classification societies consists of twelve marine classifications societies headquartered in London*”, VN_C_J)) and complementary roles during the investigations (“*together with the ship builder XX, the classification society YY will continue the thorough investigation to find the cause of the MM accident*”, VN_M_C). We consider this to constitute what we will term in Fig. 7 the ship external ecosystem and the global maritime ecosystem. The creation of a sub-corpus with Cluster 4 enabled us to understand its content further.

There are four main clusters extracted from the sub-corpus of Cluster 4. Cluster 1 corresponds to the international navigation laws and related organisations aimed at applying these regulations (‘directorate’, ‘department’, ‘republic’, ‘authority’, ‘the law’, ‘resolution’, ‘the flag state’ such as ‘Liberia’, etc.). Figure 7 will name these organisations the ‘global maritime ecosystem’. Moreover, Clusters 2, 3 and 4 refer to the ship external stakeholders that indirectly interact with her; for instance, the classification societies and the port authorities, to name a few. In Fig. 7, we will name these stakeholders the ‘ship external ecosystem’.

In a nutshell, the analysis of the sub-corpus of the main Cluster 4 leads to a differentiation of stakeholders that regulate navigation and the stakeholders that take decisions in the narrow ecosystem of the ship with potential consequences on its navigation. In Fig. 7, we will name these stakeholders the ‘ship external ecosystem’.

Cluster 5 provides an explanation of accidents related to external factors such as strong wind, bad weather, poor visibility, and high speed of the vessel (or ships close to the vessel in cases of collision).

Looking into details on the content of TS that belong to clusters 2 (technical issues), 3 (behaviours of individuals) and 5 (bad weather, poor visibility, high speed), we clearly see that these risk factors are presented as the result of the ‘fate’ even though the organisation and all stakeholders were compliant with international regulations and the safety management systems. All in all, these clusters represent 75,5% of all the content of investigation reports. The remaining Clusters 1 (the ship’s internal ecosystem) and 4 (the ship’s external ecosystem and global regulation organisations) only represent 24,5% of the total TS of the IMO investigation reports. We argue that these stakeholders’ roles, relationships, potential interdependencies, and responsibilities should be studied in more detail to improve our understanding of maritime navigation accident risk factors.

In "Discussion" section, we discuss our findings from the statistical analysis and present the maritime ecosystem framework, which is based on the five clusters that emerged from the data analysis and their in-depth analysis using IRAMUTEQ.

## Discussion

### Maritime accident risk indicators based on vessel characteristics

Looking firstly at the statistical findings exploring the characteristics of maritime vessels and severity of outcomes (deaths) of maritime accidents, some of the findings from this study were consistent with some studies but inconsistent with others. For instance, contrary to our findings, Li and Wonham (1999) found a relationship between accidents and flag states under which the ships were registered using the Lloyd’s Register of Ships database. Furthermore, using data from 2012 marine accidents in Hong Kong, Yip (2008) found that the potential for fatal accidents and injuries increased according to vessel types and types of accidents. These findings contradict our own, where our ANOVA found no differences in fatalities based on vessel types or flag states.

All in all, there is some consistency in the findings related to the age of a vessel and, to a much lesser extent, the vessel size, which is a subset of Fan et al.’s (2020) vessel condition risk category, impacting maritime accidents. By modelling the different types of accident scenarios, Fan et al. (2020) posit that ships aged 11–15 years had the lowest probability of being involved in a collision and vessels over 20 years old were more likely to be involved in a grounding. They also posit that ‘different accident types are correlated with different variable priorities. For example, ‘vessel condition’ (which incorporates size) ‘is the most important [risk influencing factor] for ‘sinking’, but the least important for ‘contact/crush’ ‘type accidents’ (ibid.:10).

Although these differences can arguably be attributed to the different techniques, methods, and data used to analyse maritime accidents, the lack of consistency in results is notable. From this, we can see that vessel characteristics as potential risk indicators for the seriousness of maritime accidents may not be very reliable. We argue that we need to go beyond macro analyses that are only based on variables that characterise ships and their context. In "Proposing new maritime accident risk indicators based on maritime ecosystem" section, we suggest that more research is needed to encompass the micro understanding of these causes and the potential inter-dependencies among the stakeholders within a broader maritime ecosystem that we will define.

### Proposing new maritime accident risk indicators based on maritime ecosystem

The literature review has shown that most causes of maritime accidents are largely related to individuals, fewer ones to organisations, and even fewer mention a larger ecosystem, even though they are acknowledged to a certain extent in accident classification reporting structures. For instance, according to Garrett and Teizer (2009: 756), the UK’s MAIB accident reporting categories provided opportunities to identify ‘external bodies liaison’ (policy and legislation), ‘company and organization’, in addition to ‘crew factors’, ‘equipment’, ‘working environment’ and the ‘individual’.

Similarly, by examining maritime accident investigation reports through a new lens, enabled by text mining software (IRAMUTEQ), the interpretation of the five clusters (Fig. 4a) and the related content of TS highlight that 75,5% of TS are related to what we call “fate risk factors” (clusters 2, 3 and 5). However, Clusters 1 (the ship’s internal ecosystem) and 4 (the ship’s external ecosystem) reveal the presence of different stakeholders who regularly interact before, during and after the maritime accidents occur. These interactions, with potential interdependence, are, by essence, highly complex.

In this paper, in coherence with the literature review, we defend the idea that the stakeholders both mostly mentioned in Clusters 1 (the relationships between the crew and its ship owner on shore) and 4 (external actors such as flag stages, classification societies, port authorities, insurance companies) generate new social, organisational and inter-organisational risk factors that are yet not considered in maritime reports. Moreover, the digitalisation of ships (Gavalas et al. 2022) not only brings new opportunities to facilitate navigation but also new technological risks, especially with the growing number of communications between stakeholders on board and onshore.

All these risk indicators extend beyond the narrow focus of the individual and the ship. However, as the direct impact of wider stakeholders at the accident site varies considerably according to their position within and beyond the boundaries of the ship, it is sometimes difficult to discern. Indeed, the stakeholders cited in Cluster 4, are so distant from the accident site, that the decisions they make are often less visible and so often go unreported in the literature and under-reported in the accident investigation reports (evidenced in cluster 4 with less than 13% of TS of all IMO reports).

These findings are in line with Hollnagel and others who recognise that factors leading to an incident or accident are not linear but are ‘combinations of mutually interacting variables’ and ‘combinations of multiple factors’ in complex systems. These need to be ‘studied, examined and understood if more accurate insights of what occurs in real-world complex socio-technical environments are to be gained’ (Klockner and Toft 2015:1736). However, Hollnagel’s (2014:117) more complex model of accident causes, incorporating a broader holistic approach that includes several functions (design, technology, maintenance), a larger temporality (before, during and after the accident) and wider scope (including individuals and organisations) still has some limitations. Namely, its application in practice and its scope that does incorporate all the actors and stakeholders within the broader ecosystem.

In order to address this limitation, this study has extended the scope of analysis of maritime accident risk indicators beyond the narrow orbit of the individual and the ship by taking a data-driven stakeholder approach. Building on the findings of this study, we propose the Accident Maritime Ecosystem (AME) Framework (presented in Fig. 7) developed by examining the narrative details of the maritime accident reports in more depth. The five clusters uncovered by the IRAMUTEQ analysis highlighted a wider ecosystem of actors involved in maritime accidents and form the foundations of the (AME) Framework based on five categories: the individuals, the ship organisation, the ship’s internal ecosystem, the ship’s external ecosystem and the global maritime ecosystem. These AME Framework are detailed as,

1. *The individual* category of actors is made up of the individual seafarers acting on board.
2. The *ship organisation* consists of all the actors involved in the decision-making, processes, procedures and actions taken related to the ship’s crew at the team level, including officers and the Captain.
3. The *ship’s internal ecosystem* comprises the crew on board and the ship owner onshore involved in the decision-making, processes, procedures and actions related to the ship.
4. The *ship’s external ecosystem* consists of all the actors involved in the navigation, management, and auditing beyond the ship’s internal ecosystems. This includes the port authorities (e.g. Vessel Traffic Services, the Port State Control), Search and Rescue organisations, flag administrations, classification societies, merchant ship companies, and salvage companies.
5. The *global maritime ecosystem* consists of the actors who enact and enforce the laws, regulations, policies and agreements that govern international maritime transport and the industry, for instance, IMO, EMSA, International Safety Agency, and International Chamber of Shipping, amongst others.

## Conclusion and recommendations

Human error continues to be highlighted as the top-cited cause of maritime accidents. The major aim of this study was to broaden and deepen our understanding of maritime accident risk factors beyond those of the human. This study adopted a data-driven approach, statistical (ANOVA) and advanced text-mining techniques (using IRAMUTEQ software) to extract more in-depth meaning from the often difficult to analyse semi-structured and unstructured narrative descriptions of the IMO accident reports. Building on the text analysis of the IMO accident data, we proposed the Accident Maritime Ecosystem Framework, which incorporates individuals, the ship organisation (on board), the internal ship ecosystem (on board and onshore), the external ship ecosystem (external factors) and the global maritime ecosystem (policies and regulations); moreover, it identifies these entities as risk factors in maritime accidents.

The AME Framework takes a stakeholder approach to accident risks in marmite shipping. It extends beyond the first layer of actors (mainly individuals and ship organisation) that commonly appear in accident reports. This framework highlights the inter-dependencies of actors that are involved in complex maritime safety decision-making and considers the associated risks related to the roles and relationships between all the intervening stakeholders at the outer layers of the maritime ecosystem. Here, we used this framework to propose more equitable and effective practical recommendations for improving safety, reducing risks, and preventing maritime accidents.

### The potential of statistical analysis with vessel characteristics and accident reports to further investigate the risks of maritime navigation accidents

Most risk factors of maritime navigation accidents are investigated through statistics with vessel characteristics and accident reports. Our results show that the different research using statistics with vessel characteristics are inconsistent. This is partly due to the development of statistics with incomplete databases, which lead to inconsistent findings and hence a lack of clarity for industry decision-makers for improving maritime safety. The development of open science databases, supported by organisations such as IMO, should improve the potential of further research in this area. Regarding maritime accident investigation reports, there is a valuable resource for learning and improving the understanding of risk indicators across the maritime ecosystem. However, as the data is unstructured, and inconsistent, and not all parts of the reports are completed, it takes significant resources to manually classify and organise the data to make sense of it. In addition, official reports are partly ambivalent: on the one hand, they claim that they are not written to identify any guilty or responsible parties; on the other hand, they indirectly influence legal decisions with potentially high financial and reputational consequences. Hence, these materials are interesting in pointing out potential causes and actors’ roles but do not develop the complex relationships among them in a long-term temporality. We argue that further in-depth qualitative and longitudinal analyses of case studies of maritime accidents, integrated with a historical approach, such as Manjarres-Wahlberg (2022), are needed to understand further the risk factors embedded in a complex set of actors that are partly interdependent similarly to an ecosystem.

This study has shown that the reporting of causes of maritime accidents focuses on a very narrow characterisation of human error as a ‘catch-all term’ that is often too vague and far too limited to provide meaningful and novel insights. Maritime reports also mention the role of leadership of the master and ship management concerning regular communication with the ship owner (Cluster 1), as well as the role of external actors (flag administration, flag states, authorities, ports, classification societies in Cluster 4) mainly regarding the respect of the safety management system and related controls. However, the roles of the actors of the ship’s internal, external and global maritime ecosystems (Cluster 5) remain blurred.

### Enlarging the study of maritime navigation accidents from ‘fate’ risk factors to a holistic and in-depth understanding of the complex relationships between business and safety in the holistic ecosystem

In order to gain a more holistic and in-depth understanding of risk factors in maritime accidents, this study explored the complex relationships between the different entities and actors within the wider maritime ecosystem and the role that they might potentially play.

#### The ship internal ecosystem: from a control perspective to a deep understanding of the role of the ship management and its relationships with the ship owner

Most risk factors highlighted to explain accidents can be related to fate factors (external factors such as the weather—Cluster 5-, technical issues of the vessels—Cluster 2—and unpredictable behaviours of individuals—Cluster 2). These findings highlight the influence of the CASMET categorisations on the structure of maritime accident reports. These categorisations were developed several decades ago, which are largely human-centric and do not fully consider other types of risks, such as the decisions made by the ship owner to the ship management or the current impact of the rapid technological advances.

Finally, accident reports point out the role of the ship’s internal ecosystem by splitting, on the one hand, the role and decisions of the leadership and management on board and, on the other hand, the decisions of the ship owner. Both leadership and management on board are mainly mentioned to report potential limitations in control of the safety management system. In principle, the managerial structure responsible for hiring, training, and putting an individual in a specific position tends to fade in favour of organizational responsibility. However, the frequency of the term ‘human error’ and the importance of the ‘fate risk factors’ (755%) in global maritime reports with several errors directly attributed to the ‘individuals’ (cluster 3–19,4% of TS) questions how far the individual responsibility is not over-represented in comparison to the organisational responsibility.

In addition, reports rarely mention the decisions made by ship owners from the shore that may endanger maritime navigation. A few reports mention navigational instructions provided by the ship owner from the shore that may explain a change in the vessel navigation decision (i.e. increase the speed), a change regarding the initially planned routes that may lead to additional risks that could have been avoided. Besides, a few reports mention that the crew’s resources were insufficient or adequate to navigate safely. We argue that further research should be developed on the effects of the decisions of ship owners, directed by business indicators, on the safety of the crew or individuals and their capacity to potentially respond to more risky situations. Finally, as the industry increasingly relies on information technologies, the potential risks to the safety of the seafarers, the vessels and the broader marine ecosystem are incalculable. Indeed, having closely examined the IMO reports, less than a handful of reports mentioned risk factors related to new information technologies—for instance, cyber-attacks.

#### The global maritime and ship external actors’ ecosystem and its impact on the actors closest to the ship

The use of IRAMUTEQ in this context helps advance the field by providing new insights into the contributing risk factors involved in maritime accidents beyond those related to the ship, how they are linked and which risk factors contribute to an accident. The AME framework proposed here conceptualised and defined the risk indicators across a broader range of responsible actors in the maritime ecosystem, namely the flag states and administration, the authorities, the ports and the classification societies. In maritime reports, these actors are mentioned without analysing their roles, relationships and the potential domino effects of this chain of actors leading to different natures of risk factors such as social, organisational and inter-organisational. Recent research is aligned with the need to further investigate the changing roles of port authorities with a potential lack of power to detain ships with technical deficiencies (Notteboom and Winkelmans 2001), the dilution of responsibilities between a fragmented supply chain of operators regarding technical audits, with multiple contradictory roles (Berg et al. 2013), the potential corruption of certain actors such as classification societies (Mahmud and Rossette 2007) and their links to ship owners as their shareholders (Baumler and François 2005), the pressure on costs leading to social and environmental risks (Sislian et al. 2016), the role and dynamics among several classification societies in the whole transportation supply chain (Fulconis and Lissilour (2021), the need to improve the transparency of the governance of ports (Brooks 2022; Manjarres-Wahlberg 2022). This framework can be used to provide a guide for maritime industry policymakers and organisations, to better understand maritime accident risk indicators related to the complex relationships of external stakeholders with new and emerging circumstances highlighting at the same time areas for improving the quality, quantity and structure of the accident data captured in official accident reports.

For instance, industry practitioners should include inter-organisational risk factors that incorporate the relationships among the different actors of the AME framework and their consequences on risk accidents. Furthermore, based on an in-depth understanding of the risk factors at the AME ecosystem level, the categorisation grids that structure maritime reports such as CASMET should be updated with the integration of new types of risk situations and new indicators to assess the level of damage from different perspectives and the respective consequences. Finally, to improve knowledge transfer and learning from maritime accidents, this study recommends an open science database to share knowledge and information and ensure that the often-reported recommendations are enacted and developed into practical and applied measures.

As with all research, there are limitations. This study has focused on a limited number of variables extracted from the IMO database because it is so time and resource intensive. Similarly, the text mining analyses are conducted on IMO reports over ten years; other official reports can also be studied. Future studies could extend this research to include additional information such as geo-locations, times, and types of accidents. Also, future studies could repeat this research using reports from other international maritime organisations to compare findings.

Future research could build empirical studies to better understand the impact of each of the entities within the maritime accident ecosystem, how they might influence each other, and the seriousness of the accident outcome. Also, further investigation is needed to explore how and where the very promising and emerging advanced machine learning and artificial intelligence techniques could be applied. However, they must be built on data that reveals the complexity of the accidents beyond the human-centric focus inherent in accident reports to avoid bias and find solutions that address risk factors within the broader ecosystem.

## Data Availability

All data generated and analysed during this study are available on request.

## Abbreviations

AME: Accident maritime ecosystem
ANOVA: Analysis of variance
CASMET: Casualty analysis methodology for maritime operations
CFA: Correspondence factor analysis
DHCA: Descending hierarchical classification analysis
EMCIP: European maritime casualty information platform
EMSA: European MARITIME SAFETY AGENCY
GISIS: Global integrated shipping information system
HE: Human error
IMO: International maritime organisation
MARPOL: MARine POLlution
SA: Similarity analysis
SOLAS: Safety of life at sea
WYLFIWYF: What-You-Look-For-Is-What-You-Find
